# Dietary Selenium Deficiency Partially Mimics the Metabolic Effects of Arsenic

**DOI:** 10.3390/nu13082894

**Published:** 2021-08-23

**Authors:** Christopher M. Carmean, Mizuho Mimoto, Michael Landeche, Daniel Ruiz, Bijoy Chellan, Lidan Zhao, Margaret C. Schulz, Alexandra M. Dumitrescu, Robert M. Sargis

**Affiliations:** 1 Division of Endocrinology, Diabetes and Metabolism, Department of Medicine, University of Illinois at Chicago, Chicago, IL 60612, USA; carmean@uic.edu (C.M.C.); landeche@uic.edu (M.L.); bchellan@uic.edu (B.C.); lzhaox@uic.edu (L.Z.); mschul35@uic.edu (M.C.S.); 2 Chicago Center for Health and Environment (CACHET), Chicago, IL 60612, USA; 3 Section of Endocrinology, Diabetes and Metabolism, University of Chicago, Chicago, IL 60637, USA; Mizuho.Mimoto@uchospitals.edu (M.M.); alexd@uchicago.edu (A.M.D.); 4 Committee on Molecular Metabolism and Nutrition, University of Chicago, Chicago, IL 60637, USA; daniel.ruiz@emory.edu; 5 Division of Epidemiology and Biostatistics, School of Public Health, Medical Scientist Training Program, University of Illinois at Chicago, Chicago, IL 60612, USA

**Keywords:** selenium, arsenic, nutrition, dietary intervention, insulin secretion, endocrine-disrupting chemicals, glucose tolerance, mouse model, metals, metabolism

## Abstract

Chronic arsenic exposure via drinking water is associated with diabetes in human pop-ulations throughout the world. Arsenic is believed to exert its diabetogenic effects via multiple mechanisms, including alterations to insulin secretion and insulin sensitivity. In the past, acute arsenicosis has been thought to be partially treatable with selenium supplementation, though a potential interaction between selenium and arsenic had not been evaluated under longer-term exposure models. The purpose of the present study was to explore whether selenium status may augment arsenic’s effects during chronic arsenic exposure. To test this possibility, mice were exposed to arsenic in their drinking water and provided ad libitum access to either a diet replete with selenium (Control) or deficient in selenium (SelD). Arsenic significantly improved glucose tolerance and decreased insulin secretion and β-cell function in vivo. Dietary selenium deficiency resulted in similar effects on glucose tolerance and insulin secretion, with significant interactions between arsenic and dietary conditions in select insulin-related parameters. The findings of this study highlight the complexity of arsenic’s metabolic effects and suggest that selenium deficiency may interact with arsenic exposure on β-cell-related physiological parameters.

## 1. Introduction

Arsenic is an abundant element that naturally contaminates the groundwater under an estimated 140 million people worldwide, predominantly as arsenite (As^3+^) [1]. Chronic arsenic ingestion via drinking water has been linked to a multitude of long-term adverse health effects, including all-cause mortality [2,3,4], cancer, insulin resistance, glucose intolerance, and diabetes mellitus (DM) [5,6,7,8,9,10,11]. For these and other reasons, the World Health Organization lists arsenic as one of the top ten chemicals of major public health concern [12]. From the perspective of metabolic health, the data linking arsenic to DM are especially troubling given that an estimated 463 million adults currently live with DM, and the fact that this number is expected to grow to a staggering 700 million by 2045 [13].

Studies in animal models have revealed arsenic’s multi-organ effects on glucose homeostasis [14,15,16,17], confirming its role as an endocrine-disrupting chemical (EDC). Though it is widely recognized that arsenic decreases β-cell function [17,18,19,20,21] and alters insulin sensitivity [22,23,24,25], the molecular mechanisms responsible for these effects, and their exact relevance to human health, remain poorly understood [18,20,26,27,28,29,30]. As such, addressing arsenic’s impact on human health mandates the identification of factors that exacerbate arsenic toxicity to identify individuals at heightened risk as well as to develop therapeutic approaches to mitigate the adverse effects of chronic arsenic exposure on human health. 

Studies of arsenic exposure have revealed that acute arsenicosis can be partially ameliorated by large, otherwise toxic doses of selenium [31,32]. This effect is thought to occur via the direct interaction between arsenic and selenium to form the compound seleno-bis(S-glutathionyl) arsinium, which is rapidly secreted in the bile [33]. In agreement with this proposed mechanism, higher blood selenium levels have been associated with reduced arsenic levels and better health outcomes in chronically exposed individuals [34,35,36]. If selenium were protective against the effects of arsenic by direct interaction with arsenic in the bloodstream, then we hypothesized that dietary selenium restriction would exacerbate the effects of arsenic throughout the body. Intriguingly, this was not the case in our model; rather, selenium deficiency itself partially recapitulated the effects of arsenic on glucose homeostasis and insulin secretory dynamics.

## 2. Materials and Methods

### 2.1. Animal Care

Male C57BL6/J mice from the Jackson Laboratory (Bar Harbor, ME) were group-housed under 14/10-h light/dark cycles at 22.2 ± 1.1 °C. At 8 weeks of age +/− 2 weeks, mice were switched from a standard irradiated mouse diet (#7912, Envigo Teklad, Madison, WI, USA) to an irradiated torula yeast-based custom diet containing either 0.1 ppm Se (Normal Control [NC], TD.96363, Envigo Teklad) or <0.01 ppm Se (Selenium Deficient [SelD], TD.92163, Envigo Teklad). Selenium supplementation in the NC diet was in the form of sodium selenite. From the start of the study, mice were provided ad libitum access to regular purified facility water or purified facility water containing 50 ppm sodium arsenite (NaAsO_2_, iAs, Sigma Aldrich, St. Louis, MO, USA). This exposure protocol has been previously shown to produce liver arsenic levels comparable to clinical observations of chronically exposed humans and other mouse models [17,18,19,20,21,22,23,24,25,26,27,28,29,30]. As previous studies have demonstrated that sex and sex steroid hormones affect susceptibility to the effects of arsenic [34,35], this study focused only on male mice. All animal protocols were approved by the Institutional Animal Care and Use Committee at the University of Illinois at Chicago (Approval number 19-198).

### 2.2. Intraperitoneal Glucose Tolerance Test (IPGTT)

After 8 weeks of arsenic exposure, mice were fasted for 6 h and then received a bolus of 2 g/kg glucose intraperitoneally. Whole blood glucose was measured from a tail bleed with a handheld glucometer (Contour Next meter with Contour Next test strips, Ascensia Diabetes Care, Parsippany, NJ, USA, range 20–600 mg/dL) at 0, 15, 30, 60, 90, and 120 min post-injection. At 0, 15, and 30 min, an aliquot of plasma was collected by tail bleed into heparinized tubes and placed on ice for later insulin determination. At the end of the experiment, heparinized blood was spun at 1500 *g* for 15 min at 4 °C. The supernatant was transferred to a fresh microcentrifuge tube and stored at −20 °C.

### 2.3. Arginine Stimulation Test

After 9 weeks of arsenic exposure, mice were fasted for 6 h and then injected intraperitoneally with 1 g/kg arginine solution. Blood samples were collected from tail bleed into heparinized tubes at 0, 2, 5, 10, and 15 min relative to the injection time for insulin determination.

### 2.4. Blood/Plasma Assays

Glucose was measured by hand-held glucometer (Contour Next meter with Contour Next test strips, Ascensia Diabetes Care, Parsippany, NJ, USA, range 20–600 mg/dL). Plasma insulin was quantified for the IPGTT using the Mouse Ultrasensitive Insulin ELISA kit according to the manufacturer’s instructions (80-INSMSU-E01, ALPCO, Salem, NH, USA, range 0.188–6.9 ng/mL). Plasma insulin was quantified for the arginine stimulation test using the Ultra-sensitive HTRF Insulin Assay (CisBio/PerkinElmer, Gif sur Yvette, France, range 0.24–8 ng/mL) according to the manufacturer’s instructions. Insulin determination was switched from CisBio to ALPCO for its enhanced sensitivity. Any technical replicates that were below the lowest standard were quantified as the lowest standard concentration divided by the square root of 2.

### 2.5. Sacrifice and Serum Collection

After 10 weeks of arsenic exposure, mice were fasted for 4 h and then euthanized by isoflurane anesthesia and exsanguination by cardiac puncture. The pancreas was resected, weighed, and then fixed in formalin for 48 h and stored in 70% ethanol. Immediately after pancreas harvest, gonadal adipose tissue, perirenal adipose tissue, and the liver were collected, weighed, and flash-frozen in liquid nitrogen then stored at −80 °C.

### 2.6. Metals Analysis

Hepatic tissue was digested by the addition of 3 mL of concentrated nitric acid, and 2 mL peroxide was added to the weighed sample in a screw cap polypropylene sample tube. The cap was tightened, and the tube placed in a water bath at 85 °C for 2 h, followed by dilution to 5% final HNO_3_. ICP-MS was performed on a computer-controlled (QTEGRAsoftware) Thermo iCapQ ICP-MS (Thermo Fisher Scientific, Waltham, MA, USA) operating in KED mode and equipped with a ESI SC-2DX PrepFAST autosampler (Omaha, NE, USA). Nickle skimmer and sample cones were used from Thermo Scientific (part numbers 1311870 and 3600812). Internal standard was added inline using the prepFAST system and consisted of 1 ng/mL of a mixed element solution containing Bi, In, 6Li, Sc, Tb, Y (IV-ICPMS-71D from Inorganic Ventures). Each sample was acquired using 1 survey run (10 sweeps) and 3 main (peak jumping) runs (40 sweeps). The isotopes selected for analysis were ^27^Al, ^51^V, ^52^Cr, ^55^Mn, ^56,57^Fe, ^59^Co, ^60,62^Ni, ^63,65^Cu, ^66,68^Zn, ^75^As, ^77^Se, ^95^Mo, ^111^Cd, ^118,119^Sn, ^121^Sb, ^206,208^Pb, and ^45^Sc, ^89^Y, ^115^In, ^159^Tb, and ^209^Bi (chosen as internal standards for data interpolation and machine stability). Instrument performance was optimized daily through autotuning followed by verification via a performance report (passing manufacturer specifications).

### 2.7. Data Analysis and Statistics

Area-under-the-curve calculations were performed by summing the areas of trapezoids formed by the glucose or insulin values measured when plotted against time. All data are represented as the mean +/− the standard error of the mean. To evaluate the effect of multiple exposures, multivariable linear regression with interaction evaluation was used to quantify the impact of diet and arsenic exposure on all continuous, single time point outcomes. The effects of arsenic and selenium deficient diet on outcomes with repeated measures were evaluated using a linear mixed model. All continuous data underwent natural log transformation due to skewness and kurtosis of the distributions. Regressions and mixed models were performed using SAS software version 9.4 (SAS Institute Inc., Cary, NC, USA). 

## 3. Results

### 3.1. Hepatic Metals Concentrations

**Figure 1:**
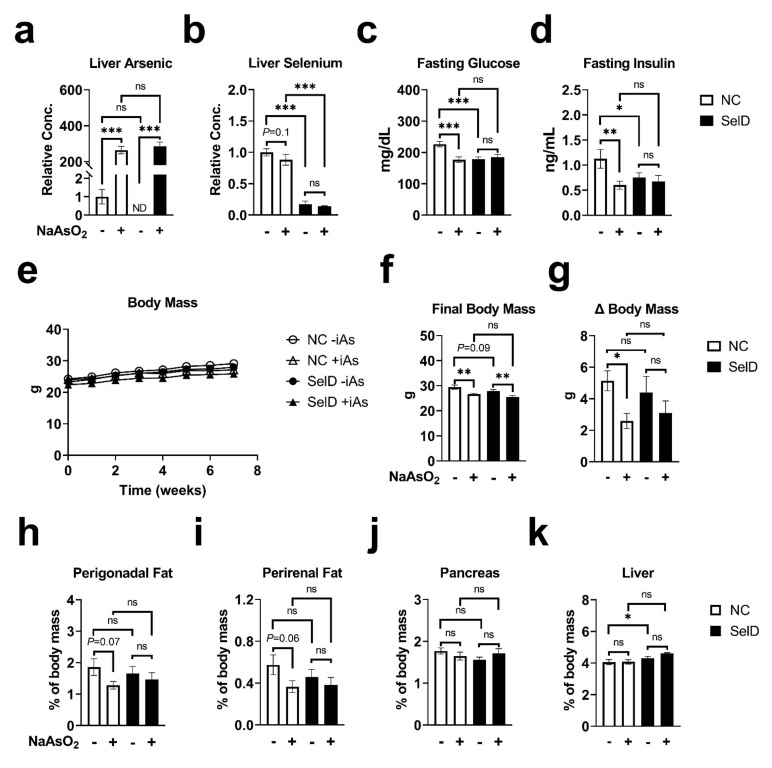
Effects of arsenic exposure and/or selenium deficiency on metabolic and organ mass characteristics. (**a**) Liver arsenic concentration normalized to controls (*n* = 4–14); (**b**) liver selenium concentration normalized to controls (*n* = 4–14); (**c**) fasting blood glucose (*n* = 11–14); (**d**) fasting plasma insulin (*n* = 10–12); Weekly body mass (*n* = 11–15); (**f**) final body mass (*n* = 10–13); (**g**) change in body mass (*n* = 10–13); (**h**) perigonadal fat mass (*n* = 11–14); (**i**) perirenal fat mass (*n* = 11–14); (**j**) pancreas mass (*n* = 10–14); (**k**) liver mass (*n* = 10–14). Data were combined from three independent cohorts. Statistics: All data were log-transformed for statistical analysis. (**a**–**d**,**f**–**k**) ANCOVA was performed to test for main effects and interactions. (**e**) Mixed-model ANOVA was used to test overall differences in body mass between groups. * *p* < 0.05, ** *p* < 0.01, *** *p* < 0.001, ns = not significant.

Mice were exposed to 50 ppm NaAsO_2_ in their drinking water for a total of 10 weeks (+iAs mice) or provided normal facility drinking water (−iAs mice) while allowed ad libitum access to either a control diet (NC mice) or a selenium-deficient diet (SelD mice). Exposure to inorganic arsenic in drinking water raised the hepatic concentration of arsenic 300-fold in both NC and SelD mice (Figure 1a). There was not a significant interaction between arsenic and SelD treatments for these measurements. Hepatic selenium was significantly reduced in both −iAs and +iAs mice fed the SelD diet versus their respective −iAs and +iAs NC controls (Figure 1b), and a trend towards decreased hepatic selenium was observed in NC mice exposed to iAs (*p* = 0.1). As with hepatic arsenic, there was not a significant interaction between arsenic treatment and selenium-deficient diet.

### 3.2. Fasting Measures of Glucose Homeostasis

There was a significant effect modification between arsenic and selenium deficiency in which arsenic significantly affected fasting glucose (Figure 1c) and insulin (Figure 1d) under normal dietary conditions, and selenium deficiency significantly decreased these parameters in the absence of arsenic. Arsenic did not further affect glucose or insulin in SelD mice. 

### 3.3. Body Measurements

Body mass was measured weekly (Figure 1c). Linear mixed-model analysis revealed significant effects of arsenic exposure and SelD diet individually on body mass with no significant interaction between these treatments. Arsenic significantly decreased body mass in both NC and SelD mice versus their −iAs controls at multiple time points (Table 1). Arsenic significantly decreased final body mass (Figure 1d) in both dietary groups. SelD diet mice showed a trend towards decreased final body mass compared to −iAs NC mice; however, this effect did not reach statistical significance (*p* = 0.09, Figure 1f). Total change in body mass was significantly decreased by arsenic exposure in NC mice, but not SelD mice (Figure 1g). SelD diet did not have a significant effect on total weight change, and arsenic exposure did not affect total weight change in SelD mice. 

Upon sacrifice, visceral fat pads were harvested along with the pancreas and liver. We observed a trend towards decreased perigonadal (*p* = 0.07, Figure 1h) and perirenal (*p* = 0.06, Figure 1i) fat pad masses as percentages of total body mass in NC +iAs mice versus NC −iAs controls. There were no significant differences in pancreas mass in any of the comparisons (Figure 1j). The livers (Figure 1k) of −iAs SelD mice (4.30% of total body weight) were slightly but significantly increased versus −iAs NC controls (4.06%).

### 3.4. Glucose Tolerance

**Figure 2:**
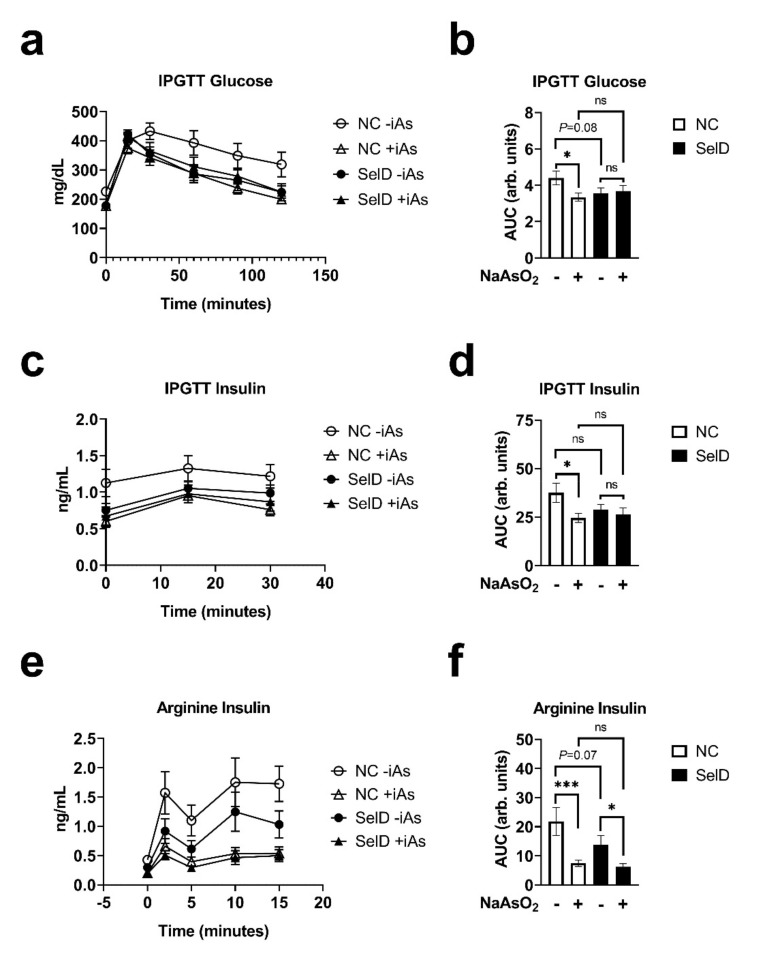
Effects of arsenic exposure and/or selenium status on dynamic metabolic parameters. (**a**) Blood glucose measured during an intraperitoneal glucose tolerance test (IPGTT) (*n* = 10–14); (**b**) area under the curve (AUC) of blood glucose values measured during IPGTT (*n* = 10–14); (**c**) plasma insulin during IPGTT (*n* = 10–14); (**d**) AUC of plasma insulin values measured during IPGTT (*n* = 10–14); (**e**) plasma insulin during arginine stimulation test (*n* = 10–11); (**f**) AUC of plasma insulin values measured during the arginine stimulation test (*n* = 10–11). Data were combined from three independent experiments. Statistics: All data were log-transformed for statistical analysis. (**a**,**c**,**e**) Mixed-model ANOVA was used to test overall differences in body mass between groups; (**b**,**d**,**f**) ANCOVA was performed testing for main effects and interactions. * *p* < 0.05, *** *p* < 0.001, ns = not significant.

To determine the effects of iAs and selenium deficiency on glucose homeostasis, we measured glucose tolerance using an intraperitoneal glucose tolerance test at the 6-week time point. Both +iAs and SelD mice showed a trend towards decreased glucose levels with a significant interaction between iAs and SelD status as determined by linear mixed-model (Figure 2a, Table 1). On individual points of the curve, both arsenic and the SelD diet significantly reduced glucose levels, especially from the 30-min time point until the end of the IPGTT. The area under the curve (AUC) during the IPGTT was significantly lower following iAs exposure in the NC group and trended lower in the SelD diet versus −iAs NC mice; however, iAs exposure did not have any further effects on IPGTT glucose AUC in SelD mice (Figure 2b).

### 3.5. Effects on Insulin Dynamics

Both arsenic exposure and SelD treatments significantly affected insulin levels during the IPGTT, and there was a significant interaction between the two treatments (Figure 2c). During the IPGTT, insulin was consistently lower in +iAs NC mice compared to −iAs NC controls (Table 1). Arsenic exposure significantly decreased insulin AUC only under NC dietary conditions (Figure 2d). Finally, to test the ability of pancreatic islets to rapidly secrete insulin in response to depolarization, which tests the effects of the amplifying pathways of insulin secretion, arginine was injected intraperitoneally and then plasma insulin was sampled. This is considered a functional in vivo measure of β-cell capacity [36]. During arginine stimulation, arsenic and diet significantly affected the insulin secretory profiles, and there was a significant interaction between arsenic exposure and diet (Figure 2e). Arsenic exposure in NC mice significantly decreased insulin secretion during this test at every time point measured (Table 1). Selenium deficiency also decreased insulin levels at 2, 5, and 15 min following arginine injection. Insulin AUC during this test revealed that arsenic decreased arginine-stimulated insulin secretion under both dietary conditions, and that there was a trend towards a decrease in insulin AUC in the −iAs SelD diet group versus NC −iAs controls (*p* = 0.07), reflecting accumulated differences that were also highlighted during curve analyses and comparisons of individual time points (Figure 2f and Table 1). The effects of arsenic on arginine-stimulated insulin secretion were not amplified by dietary selenium deficiency.

## 4. Discussion

The relative contributions of arsenic’s effects on insulin secretion and insulin sensitivity are complex, and the individual mechanisms of action remain poorly understood. Though the effects of selenium supplementation on acute arsenicosis or chronic arsenic exposure have been studied in a variety of human and animal disease models [37,38,39,40,41], to our knowledge this study is the first to directly compare the effects of combined arsenic exposure and dietary selenium deficiency on measures of glucose homeostasis.

Arsenic exposure improved glucose tolerance, decreased fasting glucose and insulin, and decreased arginine-stimulated insulin secretion. These findings largely agree with prior reports suggesting that arsenite exposure via drinking water induces two distinct effects on glucose homeostasis in mice; improvements in insulin sensitivity [23,24,25,26,27,28,29,30,31,32,33,34,35,42,43,44] and a defect in β-cell function that manifests as decreased insulin secretion [18,19,20,21,22,23,24,25,26,27,28]. Despite this consistency, it is noteworthy that other rodent model studies have reported significant decrements in glucose tolerance following a similar arsenic exposure paradigm [19,45,46], and epidemiological studies have reported worsening measures of glucose homeostasis with arsenic exposure [15]. Because glycemic control reflects a balance between insulin secretion and insulin action, the ultimate impact of arsenic exposure on global glucose tolerance may vary depending on the dose and relative effects on these two pathways, which may further depend upon the dosage, exposure context, molecular species, and/or other life history factors. Such modifying factors may also include gut microbial communities [47], which are likely influenced by diet and other housing conditions.

Selenium deficiency improved glucose tolerance, decreased fasting glucose and insulin, decreased glucose at the later time points of the IPGTT, and decreased insulin levels during the arginine stimulation test. These data favor the hypothesis that lower selenium intake may be beneficial for glucose tolerance, though this may occur in parallel with decreased β-cell capacity to secrete insulin. The results of the present study contradict recent mouse studies in which investigators reported that long-term consumption of selenium-deficient diets either did not affect, or decreased, measures of glucose tolerance [48,49,50,51]. Additionally, these results are surprising in the context of historical epidemiological findings, which broadly suggest that selenium excess or deficiency either increases or has no effect on the risk for the development of diabetes (reviewed in [52,53]). This apparent contradiction may be explained by differences in animal models, time course of selenium restriction, other elements of dietary formulation, or potential microbiome contributions to the phenotype [54]. Given that our selenium-deficient diet contained <10% of the normal selenium content used in our control diet, these results favor the hypothesis that selenium restriction in mice may be beneficial for overall markers of glucose tolerance.

Our data fit with a model in which selenium modulates glucose homeostasis via multiple metabolic effects, and therefore the mechanisms are likely to be complex. Indeed, one of the central limitations of the present study is that we are unable to define the specific mechanisms by which selenium deficiency modulates glycemic control. Future studies will benefit from specific interrogation of global insulin sensitivity to ascertain whether observed improvements in glucose tolerance arise from improved insulin action in target tissues, and whether arsenic and selenium interact in the modulation of insulin sensitivity. In addition, the current study is limited by its restriction to male mice. Further work is required to elucidate the potential sex-dependent nature of the observed relationships. Finally, given the variable impact of arsenic on glucose homeostasis in some mouse models, evaluation of additional factors that may explain this phenomenon are warranted, including characterizing the gut microbiome in studies of metabolic toxicity.

Arsenic and selenium each antagonize the other’s acute toxicity via biochemical interaction [31,32]. Based on these data, we hypothesized that the partial deprivation of tissue selenium by dietary intervention would reduce the available pool of selenium to react with circulating arsenic and thereby amplify arsenic’s metabolic effects. Under the conditions employed herein, however, we did not see that effect. Rather, we observed that selenium deficiency partially mimicked the effects of arsenic exposure. This could indicate that arsenic’s mechanism of action may be, in part, through the tissue-specific disruption of selenium availability. Though we saw only a trend towards decreased hepatic selenium, which is a major storage depot of selenium quantitatively, in arsenic-exposed mice on the control diet, it is possible that the pancreas is more susceptible to slight disturbances in selenium status and may therefore have been functionally depleted at the tissue level from either individual intervention. It is also possible that interactions between arsenic and selenium in these studies were obscured by the magnitude of their respective individual effects; perhaps lower doses of arsenic or more mild selenium deficiency may better enable deciphering potential interactions. Indeed, the extreme deficiency of selenium may represent a limitation of this study and its direct extrapolation of these findings to human populations. Future studies will be necessary to determine the extent to which dietary selenium intake and arsenic exposure antagonize, synergize, or operate through independent pathways in each of the organ systems affected in order to better understand their impact on long-term metabolic disease risk.

## Figures and Tables

**Table 1:** Summary of metabolic effects of arsenic exposure and/or selenium status (* *p* < 0.05, ** *p* < 0.01, *** *p* < 0.001).

Test	Time Point	+iAs vs. −iAs (NC Diet)	+iAs vs. −iAs (SelD Diet)	+SelD vs. NC (−iAs)	+SelD vs. −NC (+iAs)
Body Mass	0 weeks				0.05
	1 weeks		0.09		*
	2 weeks		*		0.06
	3 weeks		*		*
	4 weeks		**		*
	5 weeks	*	**		*
	6 weeks	*	**	0.08	*
	7 weeks	*	**		*
IPGTT Glucose	0 min	***		***	
	15 min				
	30 min	*		0.08	
	60 min	0.07		*	
	90 min	*		0.07	
	120 min	**		*	
IPGTT Insulin	0 min	**		*	
	15 min	*			
	30 min	*			
Arginine Stimulation	0 min	**		0.07	
	2 min	**		*	
	5 min	**		*	
	10 min	**	*		
	15 min	***	*	*	

## Data Availability

The data presented in this study are available on request from the corresponding author.
